# Boston Society of Medical Sciences

**Published:** 1871-02

**Authors:** 


### Selections.

### BOSTON SOCIETY OF MEDICAL SCIENCES.

Dr. Edes read a paper describing some observations of his confirmatory of Cohnheim's theory of inflammation.

Dr. Warren (he said) demonstrated to us last summer, some of the appearances seen in Cohnheim's experiments on the frog's mesentery; but time did not permit, on our part, of the careful observation necessary to see the essential point, that is the actual passage of a white globule through the walls of the vessels. The accumulation of white corpuscles inside the walls of the vessels and their appearance outside were not first observed by Cohnheim, but no one before him has described the passage through the walls. This phenomenon I was anxious to see for myself, and having obtained, by Dr. Warren's politeness, a supply of woorara, I set to work upon frogs.

I administered the drug, I think, to at least a dozen, keeping some of them twenty-four or forty-eight or more hours upon the stage of the microscope, and had occasion, once or twice, to inject the woorara for the second time. Into the lymph sacs of two I injected vermillion several times previous to exposing the mesentery, and observed many white corpuscles which had taken up the granules.

I was not, however, in any of these, at all satisfied that I had seen a single corpuscle pass through the wall of the vessels. The accumulation of them is very easily seen, and takes place, to some extent, within a very few minutes. Also, outside of the vessels, after a while, I could see irregular forms, some still changing, others stationary. A few times I saw cells of a form which answer very well to the description of those which have passed through the walls and are moving away from them. A similar form is often observed inside the vessels, where a white corpuscle becomes adherent, and is then pulled along by the current, so that a long, thread-like process is drawn out, and the corpuscle is anchored and swings like a buoy in a tide way.

Roloman Balogh, who denies the accuracy of Cohnheim's observations, speaks of the field being suddenly obscured by a multitude of white corpuscles, which he thinks arise from hemorrhage. I have also been troubled in this way, and have no doubt that these corpuscles arise from a hemorrhage, which, so far as I have seen, is, to a greater or less extent, almost inevitable. They swim over or under the mesentery, sometimes moved by the object glass coming in contact with the intestine, or some other accident. Why they should, as they sometimes do, separate themselves from the red corpuscles, is more than I can say; perhaps because coming from some vessel principally filled with them.

In fact, there seemed so many sources of deception, and the direct observation of what I wished to see seemed so difficult, that I was beginning to doubt the fact.

Finally, however, I was so fortunate as to see, within a short time, nine white corpuscles leave the vein within a limited space, and a portion of these I was able to make out, though less distinctly, on the outside. Three of these seemed to go through the same place, then I saw another disappear gradually in another place, two in another, and, finally, three near to the first three, though not all in the same place.

On the other side of the vein was a red corpuscle hanging half out and half in the vein, which did not change its position during the time I was observing it. Then a red corpuscle came down on the same side where the white ones had disappeared, and stuck flatwise to the wall. When I looked at this again, after a short interval, a little piece of the up stream end seemed caught in the wall of the vein, as if it had begun to go through. As to the appearance of the corpuscles outside the walls, it seemed that they were not round and distinct as within, but much more irregular and indistinct. When two or three had gone throu h at one place, it was not easy to separate them afterward. I felt no doubt, however, as to their having gone through, instead of being washed away. Amœboid movements, though I have seen them within the vein, were, in the case of the corpuscles which I saw go through, usually very slight. I did not trace any cell making its way from the wall after passing through, though, as I said, the beaker-shaped cells, with long stems, were probably doing so. I did not look very carefully for this, having satisfied myself on the more important point.

I should not have thought of offering these remarks to the

Society had not my observations and the fact that some observers deny the truth of Cohnheim's observations shown that the essential point, namely, the passage of the cells through the walls, is not a perfectly simple affair to see.

Dr. Edes also showed a preparation received from Dr. Woodward, U. S. A., intended to show the epithelium and stomata in the vascular walls.

Dr. Warren said that after a great number of experiments he had observed the entire process, from the appearance of the corpuscle floating in the current of blood to its appearance external to the vessel wall, in only two instances, although he had very frequently seen the corpuscles fixed in the wall. There were so many disturbing influences from the vast numbers of corpuscles in the field, from their rapid movement, &c., that most careful and concentrated observation was necessary to observe the entire process.

Dr. White stated that Mr. Morton, a medical student, during the past summer had watched the white blood corpuscles pass through the walls, and on one of his preparations Dr. White himself had watched one corpuscle as it passed through, and had satisfied himself that the corpuscles do pass as described by Cohnheim.

Dr. Dwight stated that Prof. Stricker considered the walls of a vessel as merely a protoplasm without openings, and that the corpuscles worked their way through this as a fish through the water.

Dr. Warren said that Recklinghausen claimed to have seen the stomata in capillaries which had been injected with nitrate of silver. Recklinghausen had also claimed to have demonstratad the existence of stomata in layers of epithelium by carefully placing the diaphragm of a guinea-pig over a cork ring and on top of this dropping a little milk; he had thus seen the globules of fat pass through the epithelial layer into the sub-epithelial cellular tissue.—*Medical and Surgical Journal.*

